# Establishing content validity for a conceptualized instrument to measure barriers to eating a healthful diet in adults: a consensus approach

**DOI:** 10.1186/s12913-020-4890-7

**Published:** 2020-01-16

**Authors:** Enia Zigbuo-Wenzler, Gayenell S. Magwood, Martina Mueller, Angela Fraser

**Affiliations:** 1 Hermannsweidstrasse 1, 8832 Wollerau, Switzerland; 2 Medical University of South Carolina, College of Nursing, 99 Jonathan Lucas Street, MSC 160, Charleston, SC 29425-1600 USA; 3 College of Agriculture, Forestry and Life Sciences, Clemson University, Clemson, SC 29634 USA

**Keywords:** Content validity, Multidimensional, Instrument, Establish, Barriers, Dietary practice, Factors, Behavior change wheel, Theoretical domains framework, Behavioral domain

## Abstract

**Background:**

A poor quality diet is a well-known risk factor for many chronic diseases. However, eating a healthful diet is not always simple as many underlying factors can impede adherence. Individuals with fewer barriers are more likely to eat a healthful diet than those who have more barriers. Accurately measuring barriers to eating a healthful diet could inform personalized interventions, particularly those aiming to prevent chronic diseases. The aim of this study was to establish content validity for selected items obtained from the National Health and Nutrition Examination Survey (NHANES) database to be considered for inclusion as items on the conceptualized Dietary Health Status (DHS) instrument, which is designed to measure barriers to eating a healthful diet in adults.

**Methods:**

The Behavioral Change Wheel hub COM-B and the Theoretical Domains Framework (TDF) were the two theoretical frameworks underpinning the development of the DHS instrument. Seven steps were conducted to create the instrument: 1) development of operational definitions for each TDF domain; 2) identification of items from the NHANES database 2011–2012; 3) screening of items to ensure inclusion of all relevant items; 4) assigning items to a theory-based domain; 5) evaluation of the items against inclusion/exclusion criteria; 6) solicitation of feedback from expert reviewers to reach consensus on inclusion into a domain; and 7) validation of items.

**Results:**

A total of 170 items representing twelve domains were identified as potential barriers to eating a healthful diet-- knowledge, optimism, beliefs about consequences, beliefs about capabilities, reinforcement, memory, attention and decision processes, environmental context and resources, social influences, emotion, behavioral regulation, health identity, and functional status.

**Conclusion:**

Expert review consultation and a consensus approach established content validity for 12 theory-based domains comprised of 170 items identified as potential barriers to eating a healthful diet. The use of these explanatory domains may: assist researchers to better understand barriers to adult dietary practices; inform the development of a screening tool that could be used in a community setting to measure barriers to eating a healthful diet; and inform individualized interventions.

## Background

Globally, a poor quality diet as compared to other risk factors was reported to be responsible for most deaths [1]. Diet is also a well-known risk factor for the onset of many chronic diseases, such as cardiovascular disease, diabetes, and stroke [2–4]. In the United States (US), diet is also a leading cause of premature deaths and disability related to chronic diseases, with 83.9% of deaths attributed to cardiovascular diseases reported to be associated with eating a poor quality diet [2].

Eating a healthful diet is not always simple. Many factors influence dietary practices, such as economic status, physical environment, social networks, and psychological and cognitive abilities. Not surprisingly, many investigators have reported that individuals who have fewer barriers tend to eat a healthful diet more frequently than those who experience more barriers [5–10] supporting the need to measure dietary barriers. Multiple definitions exist for “diet” ranging from all foods and drinks eaten to specific food intake that provides adequate nutrients to sustain one’s health. For the purpose of this study, “diet” refers to all foods and drinks eaten.

Given the strong link between eating a healthful diet and chronic disease, we assert that healthcare practitioners need to identify barriers to eating a healthful diet at the individual level. An instrument that measures barriers to eating a healthful can serve two broad purposes. First, it could identify individuals who might be at an increased risk for chronic diseases. Secondly, it could be used to inform individualized interventions to prevent or reduce one’s risk of chronic diseases.

Ideally, the National Health and Nutrition Examination Survey (NHANES), a population-based survey administered annually in the United States, could be used to measure barriers to eating a healthful diet. It is comprehensive, consisting of five core sections, each containing multiple components with multiple items. However, NHANES in its entirety is too complex to administer in a community setting. Even so, individual items could be extracted from the NHANES instrument to construct a shorter instrument. Another alternative would be to use one of two validated nutrition assessment tools, commonly used in community settings – the Mini Nutrition Assessment and the Determine Your Nutritional Health checklist. Both were specifically designed to screen for risk factors associated with malnutrition in older adults, hence in their present form have limited use in the general adult population [11, 12]. Other instruments that measure diet quantity, quality, access, and availability at the individual level exist but to our knowledge no validated instruments measure the multidimensional nature of barriers to eating a healthful diet. For the purpose of this study, “multidimensional” refers to separate dimensions comprising multiple factors that may influence dietary practices, which is similar to Edwards’s definition [13]. The literature shows that eating a poor quality diet is almost always influenced by multiple factors within four dimensions -- physical, psychological, cognitive, and/or social [14–16]. Descriptions of key concepts used in this study are provided in Table 1.

**Table 1:** Description of key concepts

Key concept	Description
Dietary Practice	An individual’s choices in food consumption
Diet	The types of food one eats
Dietary Health Status (DHS)	The concept of an instrument to measure the multidimensional nature of individual dietary practices
Multidimensional	Multiple, but separate, behavioral dimensions comprising multiple factors that may influence a behavioral concept (dietary practice)

### Study aim

The aim of this study was to establish content validity for selected items obtained from the NHANES database to be considered for inclusion as items on the conceptualized Dietary Health Status (DHS) instrument, designed to measure barriers to eating a healthful diet in adults.

## Methods

This study was exempted as human subject research by the Institutional Review Board of the Bioethics Committee of the Medical University of South Carolina.

### Conceptualized instrument

The DHS instrument is conceptualized as including three dimensions -- Dietary Access, Dietary Quality, and Dietary State-Of-Mind -- based on the assumption that measuring all three could result in a more comprehensive picture of barriers to eating a healthful diet. The three dimensions were anticipated to be further subdivided into eight domains (see Table 2 for details). The eight domain subscales could be scored individually as well as collectively to yield a total DHS score. The scoring system for DHS would hypothetically be based on a 100-point scale with 0 (more barriers to eating a healthful diet) to 100 (less barriers to eating a healthful diet).

**Table 2:** DHS dimensions abbreviation and description. Description of the dimensions the conceptualized instrument is comprised of. These include 3 overarching conceptualized dimensions presumed to further subdivide into eight sub-dimensions

Terms	Description
Whole Instrument	
Dietary Health Status (DHS)	Comprises 8 sub-dimensions
Three overarching dimensions	
Dietary Access (DA)	Comprises individuals’ financial resources, food security status, and access to local and federal governmental nutritional/food assistance programs, as well as non-governmental resources through community efforts that might influence diet.
Dietary Quality (DQ)	Comprises type of diet consumed, habits that might influence the quality of diet consumed [substances/drugs (i.e. illicit and non-illicit, alcohol, nicotine, marijuana)], practices such as eating out or carryout, and physical functioning.
Dietary State-Of Mind (DS)	Comprises an individual’s perception and knowledge about diet, health, and disease, as well as his/her mental and emotional functioning that reflect the state-of-mind regarding diet in general.
Eight Subdimensions	
Dietary Food Status (DFS)	
Dietary Resource (DRS)	
Dietary Quality Sub (DQS)	
Dietary Quantity (DQN)	
Dietary Habits (DHB)	
Dietary Perception (DP1)	
Dietary Knowledge (DKW)	
Dietary Psyche (mental state)(DP2)	

### Theoretical framework

Two theoretical frameworks underpinned the process for identifying and assigning items -- Behavioral Change Wheel (BCW) and the Theoretical Domains Framework (TDF) [17, 18]. The BCW framework consists of three dimensions: sources of behavior, called the hub.

COM-B, intervention functions, and policy categories [17]. The hub COM-B recognizes that behavior is part of an interacting system involving three components: capability, opportunity, and motivation. Because the aim of this study was to identify barriers to eating a healthful diet at the individual level, the only BCW dimension that was used in this study was the hub COM-B. The TDF, the other theoretical framework used, combines behavioral change theories into one master framework to help investigators identify factors influencing behavior change [17, 18]. TDF was first developed by Michie and colleagues [19] then refined in 2012 by Cane et al. [18]. It includes 14 domains associated with behavior change: knowledge, skills, social/professional role and identity, beliefs about capabilities, optimism, beliefs about consequences, reinforcement, intentions, goals, memory, attention and decision processes, environmental context and resources, social influences, emotion, and behavioral regulation [18] (Table 3). Investigators have used the TDF to develop theory-based instruments to measure potential factors influencing human behavior as well as to guide the design of interventions [20–22].

**Table 3:** Theoretical Domains Framework (TDF) 14 domain version, domains description, and 84 theoretical constructs. Cane et al. [18] definition of the Theoretical Domains Framework (TDF) 14 domains a list of the theoretical constructs comprising each domain

Theoretical Domain	Cane et al., 2012 [18] Domain Description	Theoretical Construct
1. Knowledge1.1.	An awareness of the existence of something	1. Knowledge (including knowledge of condition /scientific rationale)
		2. Procedural knowledge
		3. Knowledge of task environment
2. Skills1.1.1.1.1.1.	An ability or proficiency acquired through practice	4. Skills
		5. Skills development
		6. Competence
		7. Ability
		8. Interpersonal skills
		9. Practice
		10. Skill assessment
3. Social/professional role and identity1.1.1.1.1.1.1.	A coherent set of behaviors and displayed personal qualities of an individual in a social or work setting	11. Professional identity 12. Professional role
		13. Social identity
		14. Identity
		15. Professional boundaries
		16. Professional confidence
		17. Group identity
		18. Leadership
		19. Organizational commitment
4. Beliefs about capabilities1.1.1.1.1.1.1.	Acceptance of the truth, reality, or validity about an ability, talent	20. Self-confidence
		21. Perceived competence
		22. Self-efficacy
		23. Perceived behavioral control
		24. Beliefs
		25. Self-esteem
		26. Empowerment
		27. Professional confidence
5. Optimism1.1.1.	The confidence that things will happen for the best	28. Optimism
		29. Pessimism
		30. Unrealistic optimism
		31. Identity
6. Beliefs about consequences1.1.1.1.	Acceptance of the truth, reality, or validity about outcomes of a behavior in a given situation	32. Beliefs
		33. Outcome expectancies
		34. Characteristics of outcome expectancies
		35. Anticipated regret
		36. Consequents
7. Reinforcement1.1.1.1.1.1.	Increasing the probability of a response by arranging a dependent relationship, or contingency	37. Rewards (proximal/distal, valued/not valued, probable/improbable)
		38. Incentives
		39. Punishment
		40. Consequents
		41. Reinforcement
		42. Contingencies
		43. Sanctions
8. Intentions1.1.	A conscious decision to perform a behavior or a resolve to act in a certain way	44. Stability of intentions
		45. Stages of change model
		46. Trans theoretical model and stages of change
9. Goals1.1.1.1.1.	Mental representation of outcomes or end states	47. Goals (distal/proximal)
		48. Goal priority
		49. Goal/target setting
		50. Goals (autonomous/controlled)
		51. Action planning
		52. Implementation intention
10. Memory, attention and decision processes1.1.1.1.	The ability to retain information, focus selectively on aspects of the environment, and choose between two or more alternatives	53. Memory
		54. Attention
		55. Attention control
		56. Decision making
		57. Cognitive overload/tiredness
11. Environmental context and resources1.1.1.1.1.	Any circumstance of a person’s situation or environment that discourages or encourages the development of skills and abilities, independence, social competence	58. Environmental stressors
		59. Resources/material resources
		60. Organizational culture /climate
		61. Salient events/critical incidents
		62. Person x environment interaction
		63. Barriers and facilitators
12. Social influences1.1.1.1.1.1.1.1.1.1.	Those interpersonal processes that can cause an individual to change their thoughts, feelings, or behaviors	64. Social pressure
		65. Social norms
		66. Group conformity
		67. Social comparisons
		68. Group norms
		69. Social support
		70. Power
		71. Intergroup conflict
		72. Alienation
		73. Group identity
		74. Modelling
13. Emotion1.1.1.1.1.1.	A complex reaction pattern, involving experiential, behavioral, and physiological elements, by which the individual attempts to deal with a personally significant matter or event	75. Fear
		76. Anxiety
		77. Affect
		78. Stress
		79. Depression
		80. Positive/negative affect
		81. Burn-out
14. Behavioral regulation1.1.	Anything aimed at managing or changing objectively observed or measured actions	82. Self-monitoring
		83. Breaking habit
		84. Action planning

**Fig. 1:**
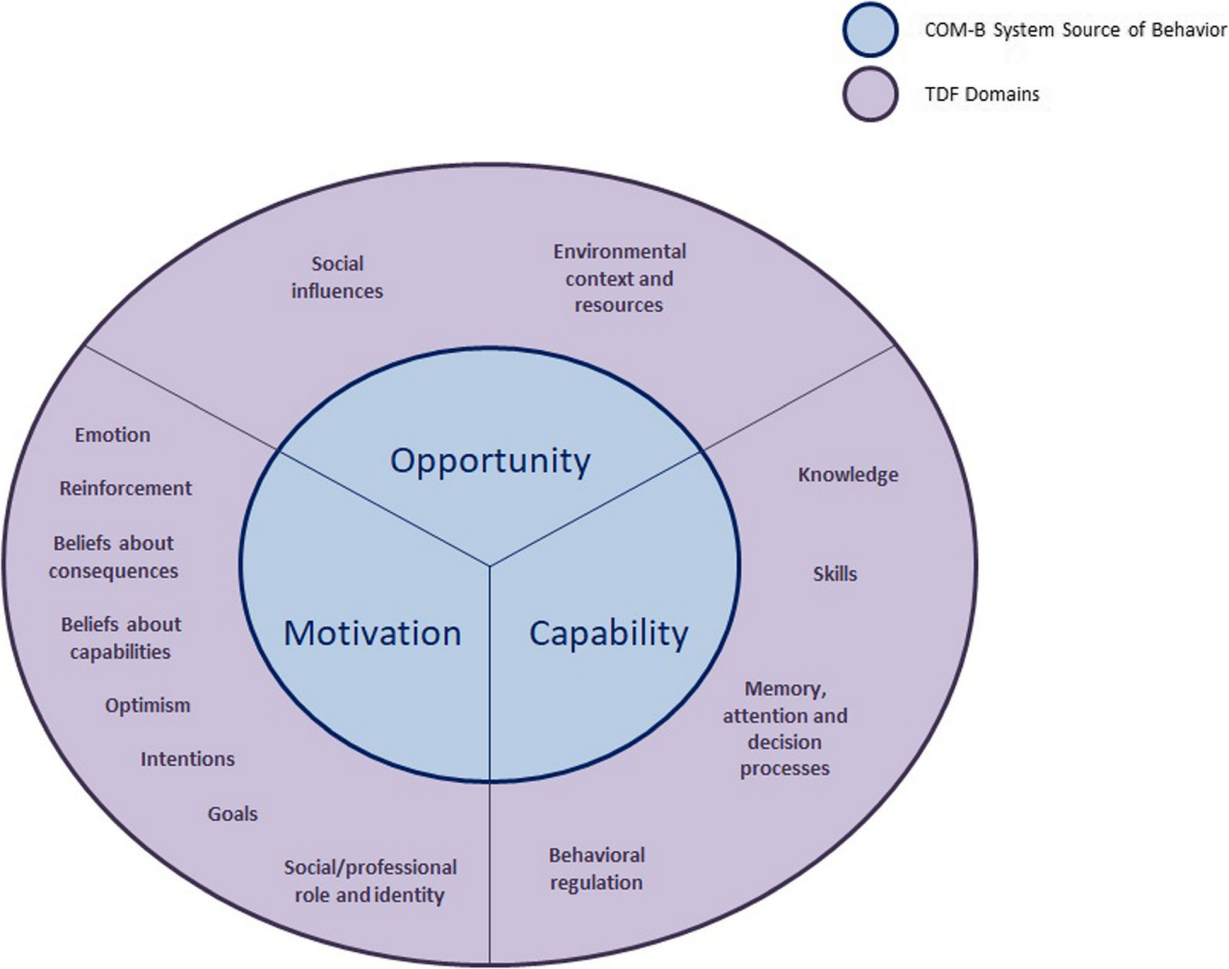
Relationship between TDF domains and COM-B; Diagram illustrates how TDF is an elaboration of COM-B; such that each domain of the TDF relates to a COM-B

Both BCW hub COM-B and TDF are interconnected as the TDF is an elaboration of the COM-B, such that each TDF domain relates to a COM-B component. Together both provided a theory-based systematic approach to: (1) select items from the NHANES datasets that could be measures of barriers to eating a healthful diet and (2) assign those items to a mutually-exclusive theory-based domain. The relationship between the COM-B and the TDF domains is illustrated in Fig. 1**.**

### Datasets and target population

NHANES data is a nationally representative sample of non-institutionalized U.S. residents. NHANES data were chosen because it has been a primary source of comprehensive health and nutrition data at the national level for nearly half a century [23]. Moreover, NHANES data is widely used by researchers to study the relationship between diet, nutrition, and health of the U.S. population. We chose to use data from cycle years 2011–2012 because at the time of this study, those datasets included the most current 24-h dietary recall data, converted into their appropriate amounts and Food Pyramid components (i.e., converted amounts of fruit, vegetables, grains, protein foods, dairy, oils, added sugars, solid fats, and alcoholic drinks).

NHANES, cycle years 2011–2012, categorizes data into five sections: demographics, examination, laboratory, questionnaire, and dietary, which includes 24-h dietary recall data. The 2011–2012 NHANES cycle years included 13,431 individuals. Of those, 9756 completed the survey interview, and 9338 had health exams [24]. A total of 3705 participants met the case inclusion criteria: 1) participants had to be > 20 years as it was assumed younger individuals might not have full autonomy over their diet and 2) had relevant data collected during in-home interviews and health examination that fit the 14 theory-based TDF domains influencing behaviors and the conceptualized dimensions of the DHS instrument. Furthermore, individuals, who met the case inclusion criteria, were excluded if any demographic and clinical data relevant to this study were missing or if they were reported to be pregnant. Pregnant women may have atypical dietary patterns.

### Expert reviewers

Six expert reviewers were identified, two declined because of time constraints. The four reviewers who agreed to participate had expertise in the behavioral, social, and/or nutritional sciences. Reviewers were tasked with providing their expert opinion on a proposed list of items assigned to one of the 14 theoretical domains that comprise the TDF. The research team -- principal investigator and three health researchers -- reviewed and validated the findings and responses from the expert reviewers.

### Procedure

**Fig. 2:**
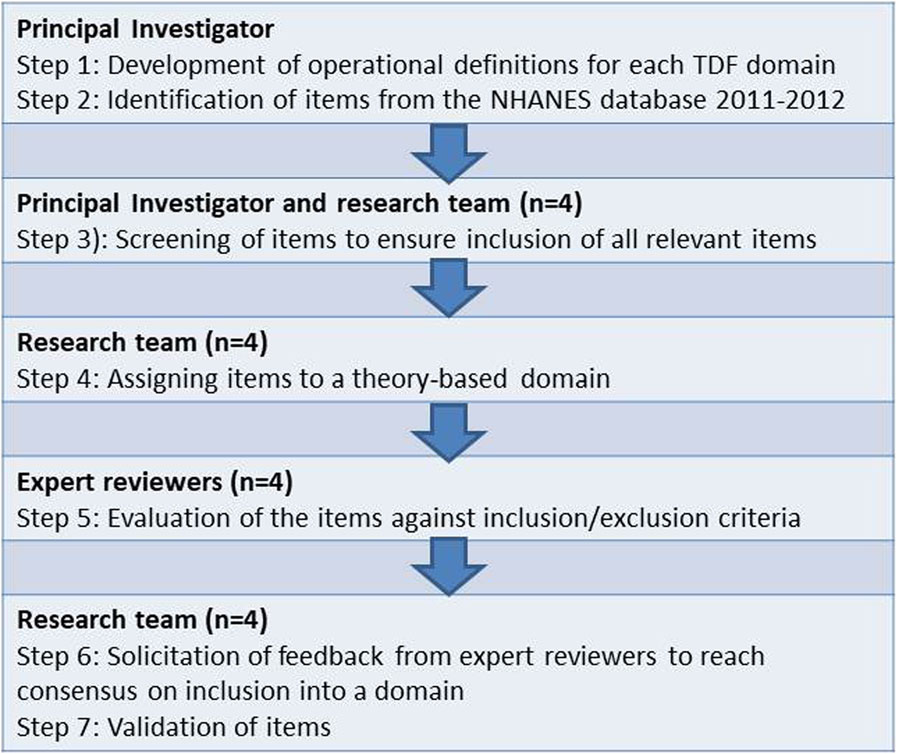
Procedural Tasks; Flow chart to illustrate procedural tasks to establish content validity for the Dietary Health Status instrument

The study was conducted between May and October 2017 (Fig. 2). Seven steps were conducted: 1) development of operational definitions for each TDF domain; 2) identification of items from the NHANES database 2011–2012; 3) screening of items to ensure inclusion of all relevant items; 4) assigning items to a theory-based domain; 5) evaluation of the items against inclusion/exclusion criteria; 6) solicitation of feedback from expert reviewers to reach consensus on inclusion into a domain; and 7) validation of items. Detailed records were kept of all meetings.

#### Step 1: development of operational definitions for each TDF domain

Operational definitions for the 14 TDF domains were generated based on the TDF domain description and the theoretical constructs that make up each theoretical domain. Refined operationalized definitions were then reviewed by the research team. Feedback from the research team on how well the operational definitions captured the domains as related to this study’s dietary focus resulted in refinement of domain definitions (Table 4). The final operational definitions were used to identify items from the NHANES database and to assign each item to one of the 14 TDF domains.

**Table 4:** Domains, descriptions and study operational descriptions. 10 TDF domains and two new created domains captured by NHANES 2011–2012 items and four of the 14 TDF original domains not captured by NHANES items

Domain	Domain Description	Study Operational Description
Knowledge^a^	Awareness of the existence of something	Awareness of the dietary guidelines, their general health and health risks factors and the benefits of sports and recreational activities
Beliefs about capabilities^a^	Acceptance of the truth, reality, or validity about an ability, talent, or facility that a person can put to constructive use	An individual’s belief about self-confidence, control, or performance concerning making appropriate dietary choices, staying healthy and engaging in sports and recreational activities
Beliefs about consequences^a^	Acceptance of the truth, reality, or validity about outcomes of a behavior in a given situation	An individual’s subjective rating of his/her general health, diet, and weight and his/her belief about the outcomes of making appropriate dietary choices, staying healthy and engaging in sports and recreational activities
Reinforcement^a^	Increasing the probability of a response by arranging a dependent relationship, or contingency, between the response and a given stimulus	Internal or external responses to a person’s behavior that affect the likelihood of making appropriate dietary choices, staying healthy and engaging in sports, fitness and recreational activities
Memory, attention and decision processes^a^	The ability to retain information, focus selectively on aspects of the environment, and choose between two or more alternatives	The ability to retain information concerning diet and health and to be able to focus on making appropriate dietary and health choices
Environ-mental context and resources^a^	Any circumstance of a person’s situation or environment that discourages or encourages the development of skills and abilities, independence, social competence, and adaptive behavior	Any characteristics of the socio-political context, organization, and the person that discourages or encourages a person to make appropriate dietary choices, stay healthy and engage in sports and recreational activities
Social influences^a^	Those interpersonal processes that can cause an individual to change their thoughts, feelings, or behaviors	An individual’s association with people and situations in society that dictates the way he/she thinks about things that might affect his/her diet, health, and sports and recreational activity level
Behavioral regulation^a^	Anything aimed at managing or changing objectively observed or measured actions	All the things a person does concerning their diet, health and sports and recreational activities
Optimism^a^	The confidence that things will happen for the best, or that desired goals will be attained	An individual’s confidence that things will happen for the best; never give up hope or look at the bright side of life
Emotion^a^	A complex reaction pattern, involving experiential, behavioral, and physiological elements, by which the individual attempts to deal with a personally significant matter or event	A subjective psychophysiological experience that might affect a person’s likelihood of making appropriate dietary and health choices, and engaging in sports and recreational activities
Skills ^a, b^	An ability or proficiency acquired through practice	The competence or capacity that help a person routinely manage otherwise his/her diet and health in a productive manner, making appropriate dietary choices, staying healthy, and engaging in sports and recreational activities
Social/professional role and identity^a, b^	A coherent set of behaviors and displayed personal qualities of an individual in a social or work setting	A coherent set of dietary and health promotion behaviors and displayed personal qualities of an individual in a social setting
Intentions^a, b^	A conscious decision to perform a behavior or a resolve to act in a certain way	Readiness/commitment to make healthy dietary choices, stay healthy and engage in sports and recreational activities
Goals^a, b^	Mental representation of outcomes or end states that an individual wants to achieve	An aim or an objective a person wants to achieve concerning their diet and health
^c^Health Identity	**New domain created; not part of TDF**	A person sense of self/identity in view of a health characteristic that he/she may have to identify with or has identified with
^c^Functional Status	**New domain created; not part of TDF**	Any functional limitations caused by long-term physical, mental, and emotional problems or illness that impact an individual’s ability to make appropriate life choices and to engage in activities that promote a healthy lifestyle

^a^TDF original 14 domains are the first 14 domains.

^b^Four of the 14 TDF original domains not captured by NHANES 2011–2012 items

^c^Two newly created domains to assign the items for which no TDF domain existed

#### Step 2: identification of items from the NHANES database 2011–2012

**Fig. 3:**
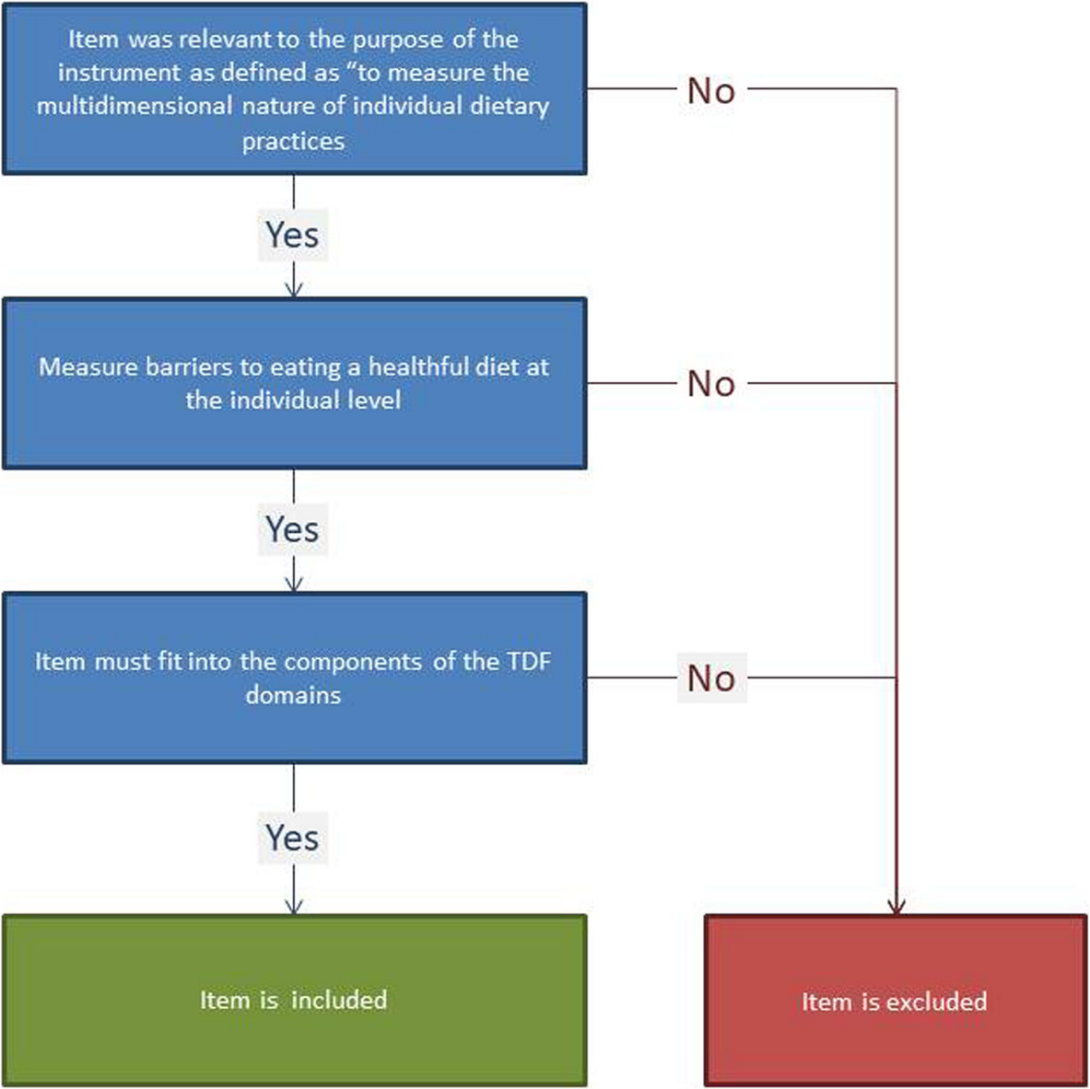
Item Identification; Flow chart to identify items based on inclusion/exclusion criteria

Initially, all five sections (demographics, examination, laboratory, questionnaire, and dietary, including 24-h dietary recall data) of the 2011–2012 NHANES data were explored to identify items that the research team believed might be a barrier to healthful dietary practices. The three inclusion criteria were: (1) item was relevant to the purpose of the instrument as defined as “to measure the multidimensional nature of individual dietary practices; (2) measure barriers to eating a healthful diet at the individual level; and (3) the item must fit into the components of the TDF domains. Items that did not meet all three inclusion criteria were excluded. The NHANES 2011-2012 datasets had a total of 148 data files. Of the 148 data files examined by the principal investigator, 133 were used to identify items to assess barriers to eating a healthful diet. The remaining 15 files did not contain data relevant to populating the DHS instrument so were not considered. An Excel spreadsheet containing names of the 148 data files and corresponding items was created. After a thorough review of each item (*N*= 3948), each item was labeled “include” or “exclude” by the principal investigator (Fig. 3).

#### Step 3: screening of the selected items to ensure no relevant items were overlooked during the initial stage

This procedure was performed to ensure no items in the NHANES datasets were improperly excluded during the initial selection. After the initial review process of identifying items from the NHANES data files and creating the excel spreadsheet item labeled “relevant” or “not relevant,” the spreadsheet was presented to the research team. The research team provided their expert opinion as to: 1) whether an item belonged to the list of identified items that could potentially measure barriers to eating a healthful diet and 2) modify the label of an item as “include” or “exclude” if necessary: (1) item was relevant to the purpose of the instrument as defined as “to measure the multidimensional nature of individual dietary practices; (2) measure barriers to eating a healthful diet at the individual level; and (3) the item must fit into the components of the TDF domains. The principal investigator initially extracted 76 based on this study inclusion criteria.

Group feedback resulted in expanding the number of items from 76 to 170; the principal investigator and one research team member extracted the additional 96 items from six supplementary data files from the initially five sections (demographics, examination, laboratory, questionnaire, and dietary, including 24-h dietary recall data) explored, increasing the number of NHANES data files where items were identified from 18 to 24. In a final review, the research team reached a consensus on “include” or “exclude” items.

#### Step 4: assigning items to a theory-based domain

Subsequent to identifying the preliminary list, the 170 items were first assigned to one of the three COM-B (capability, motivation, and opportunity) components based on the NHANES item description. This procedure categorized the items into three broad groups that captured their meaning to assists the process of further assigning the items into their expanded 14 TDF domains. After evaluation by the research team, each of the 170 items assigned to one of the three COM-B components were then assigned exclusively to one of the 14 theoretical domains of the TDF based on the study inclusion/exclusion criteria for identifying items from the NHANES files and assigning items to a theory-based domain. Furthermore, the 24 NHANES data files from which each item was extracted were reviewed to clarify if items were properly assigned to each domain. This step allowed the research team to expand the COM-B into highly specific domains as each of the 14 TDF domains relates to one of the COM-B according to Michie and colleague [17, 18].

#### Step 5: evaluation of the items assigned to each TDF domain to determine if items met the construct operational definition

Following item assignment to one of the 14 TDF domains, items within each of the domains were independently evaluated by the research team for coherence, exclusivity, and fit to the assigned domain based on: (1) specific description of the TDF domain; (2) theoretical constructs that made up the TDF domain; (3) operationalized definition of each TDF domain; and (4) description of each of NHANES item. This process was completed in two consecutive meetings during which consensus by all research team members was reached.

#### Step 6: solicitation of feedback from expert reviewers to get consensus on fit of items within an assigned domain

Following the preliminary assignment of items to the 14 theoretical domains, expert reviewers independently evaluated the list of items in each domain. The item fit within a domain was evaluated based on: specific description of the TDF domain; theoretical constructs that made up the TDF domain; principal investigator’s operationalized definition of each TDF domain; and description of each of NHANES item. Reviewers were asked to: provide their expert opinion as to whether an item belonged to the domain it was preliminarily assigned and, if necessary, re-assign the question to another domain for a better fit. Reviewers were allowed to re-assign an item only to one other domain to ensure that all items were mutually exclusive (for detailed task instructions and task spreadsheet for expert reviewers, see Additional files 1 and 2).

#### Step 7: validation of items

All reviewer responses were reviewed and summarized. The research team addressed items that did not belong as well as re-assignment of items to another domain. After two consecutive meetings, consensus was reached by the research team resulting in a final list of items assigned to10 of 14 TDF domains and two newly formed domains which captured the items four TDF domains (social/professional role and identity, goal, intention, and skills) could not.

## Results

Of the 148 data files extracted from NHANES, cycle years 2011–2012 datasets 24 files contained items included in this study. The NHANES 24-h dietary recall data file yielded 82 items of which 48 were included. A total of 170 items were included. Among those items, 169 were individual items and one item represented the composite 24-h dietary recall item calculated from a set of 48 items.

A total of 170 items were assigned to the three components that comprise the COM-B system and the 14 TDF domains. In the COM-B system, 99 items assigned to the capability component, 28 opportunity, and 43 motivation. For the TDF domains, the 170 items were assigned exclusively to one of the 14 domains as follows: knowledge (3), skills (4), social/professional role and identity (12), optimism (10), beliefs about consequences (5), reinforcement (7), intentions (1), goals (1), memory, attention and decision processes (13), environmental context and resources (15), social influences (13), emotion (7), and behavioral regulation (79). Only one TDF domain, the “beliefs about capabilities”, could not be represented by any of the items.

Evaluation and discussion regarding whether items accurately met the inclusion criteria for each domain led to further refinement. An initial concern of the research team was that four operational definitions were ambiguous, such as unclear distinctions among the domain definitions (e.g., goal, intention, optimism, and emotion domain), which led to items not being a good fit to a specific domain. Consequently, the operational definition for those 4 TDF domains was refined, and items were reassigned to another domain, if needed. Additionally, concerns were raised regarding: similarities among items assigned to the “skill” and “behavior regulation” domains, resulting in reassigning the items from the skill domain to the behavior regulation domain, thereby, eliminating the skill domain; items assigned to the “social/professional role and identity” domains were not a good fit with this domain or with any of the other 13 TDF domains. In addition, distinctions among the domains goal, intention, optimism, and emotion were ambiguous and required further clarification; for 26 of 170 items, none of the TDF domain was a good fit.

After reviewing the definitions for each of the 14 TDF domains, TDF theoretical constructs, the operationalized definition of each TDF domain, and description of each of NHANES item, the research team agreed that the skills, social/professional role and identity, goal and intention domains be eliminated and two new domains included. The two new domains, “health identity” and “functional status” were created to capture the items for which no TDF domain existed. After the domains were refined the research team agreed that the items accurately reflected their underlying domain. Overall, a total of 12 domains (knowledge, optimism, beliefs about capabilities, beliefs about consequences, reinforcement, memory, attention and decision processes, environmental context and resources, social influences, emotion, behavioral regulation, health identity and functional status) were validated, including two new domains (Table 4). The four TDF domains “social/professional role and identity, goal, intention, and skills” were not captured by NHANES items (Table 4). Additionally, Table 5 presents 12 validated domains with all the items selected from the NHANES 2011–2012 database in their complete form (see Additional file 3 for details).

## Discussion

This study was an important step that established content validity for items considered for inclusion on the conceptualized DHS instrument, which could hypothetically be used as a screening tool to measure barriers to eating a healthful diet in adults.

Diet plays a crucial role in health promotion and chronic disease prevention because diet can be changed or modified. Therefore, diet-related chronic diseases are among the most preventable [25]. However, adhering to a healthful diet may not be simple, because multiple intrinsic and extrinsic factors can be barriers to implementation of healthful dietary practices [25, 26]. These factors intertwine and are rarely one-dimensional [25, 26]. Additionally, how much each factor explains or influences dietary practices is highly individualized. Identifying barriers to healthy diet could inform the development of targeted health promotion and chronic disease prevention interventions. Cane and colleagues validated 14 TDF domains to guide researchers/developers in identifying and understanding potential factors that influence behavior change [18]. Therefore the TDF was used to ensure that all theory-based domains potentially influencing dietary practice are captured by the conceptualized DHS instrument proposed in this study in order to assess the multidimensional nature of dietary practices. Our result is inclusive of 12 theory-based domains influencing dietary practices; 10 TDF domains and 2 new domains created by the study researchers. An ideal screening instrument should include all dimensions that might potentially influence behavior change. Therefore, the items that make up each of the resulting 12 domains will be considered as items on the DHS instrument. An appropriate instrument based on a theory of behavior change that can accurately identify dietary barriers, could inform personalized interventions, particularly those that center on prevention of chronic diseases.

Two new domains that were not part of the original 14 TDF domains were created -- “health identity” and “functional status.” The “health identity” domain was defined by the researchers as an individual’s sense of self/identity in view of a health characteristic with which he/she may have to identify or has identified. However, merely being told one has a risk factor or a disease does not mean that one has fully integrated this into one’s identity; illness can either take a hold of an individual’s life partially or completely [27]. In addition, Karnilowicz states that culture plays an influential role on an individual’s sense of control and self-belief when it comes to illness or disease [28]. In particular, an individual goes through necessary shifts in identity to adapt to living with a life altering illness compared to what life was for him/her prior to the illness [28]. The “functional status” domain is defined by the researchers as any functional limitations caused by long-term physical, mental, and emotional problems or illness that impact a person’s ability to make appropriate life choices and to engage in activities that promote a healthy lifestyle.

This study provides a comprehensive process using expert review consultation to establish content validity for a conceptualized instrument that can be used by health care practitioner in the community to assess potential barriers to adult dietary practice. Additionally, this study provides the classification for questions that can be considered for inclusion as items on the instrument in 12 theory-based domains that have been demonstrated to influence individuals’ behavior. Our behavioral domains contain questions that may be key barriers to healthy dietary practices in adults. Moreover, the knowledge gained from this research may have implications for practice, education, and policy and thus informing a comprehensive approach to understanding barriers to healthy diet in adults. Regarding practice, the results can be used to inform the development of a practical screening tool that can be administered in community settings to measure barriers to healthy diet so that effective individualized interventions can be prepared. Regarding education, the results may lead to intervention training for nutrition and health assistance program developers, ensuring a multidimensional approach is used in intervention development and implementation. For example, training seminars/workshops may be developed to understand the multiple needs of individuals at increased risk for diet sensitive chronic diseases and how to intervene using a multidimensional approach. The results may be used by nutrition education curriculum developers to identify barriers that need to be identified in a curriculum for a specific target audience and might also be used by public health agencies. Regarding policy, the results may help administrators in nutrition and health organizations to identify adults with increased risk for diet sensitive chronic diseases, better understand the multiple needs of these individuals, and develop strategies that address barriers to healthy diet multidimensionally.

## Limitations

This study had several limitations to consider. The TDF framework was originally developed for implementation research by health professionals so the TDF framework fit, in the context of our population, might not be ideal for instrument development. Cane and colleagues suggested that 14 domains are necessary to analyze influences on behavior, because these domains have been validated to be influenced by potential barriers to behavior change [18]. However, this study used an existing dataset, not collected for the purpose of this study, and therefore available data were not comprehensive leading to four domains of the TDF not being represented within the NHANES datasets. Although all 14 domains may be relevant, only 10 could be represented in this exploratory study because no item considered relevant to these four domains existed within the NHANES datasets. While potentially useful, these domains may not comprehensively identify all factors that influence dietary practices, as not all TDF domains were captured by the NHANES datasets. Nevertheless, the resulting 10 TDF domains and two additional domains were well represented. Moreover, although the validated items were pulled directly from the NHANES quantitative questionnaire, we intend to further develop the instrument using a qualitative approach in a future study. This approach for the conceptualized DHS instrument would not only provide customization of the items for its intended purpose; thus allowing for the incorporation of the four TDF domains that were not captured by the NHANES pre-existing quantitative questionnaire, but would also elicit deeper insights from participants through the refinement of the items. This study reports on how content validity was established for a conceptualized instrument DHS using an expert review process. Factor analysis was then used to assess validity and reliability as determined by Cronbach’s alpha values. Results of the factor analysis are reported in detail elsewhere [29].

## Conclusions

Expert review consultation and a consensus approach, as described in this manuscript, were critical to establishing content validity for 12 theory-based domains comprised of items identified as potential barriers to adult dietary behavior. The use of these domains may: (1) assist researchers seeking to identify barriers to adults’ dietary practices for a greater understanding; (2) inform the development of a screening tool that can be used to measure the multidimensional nature of barriers to adult dietary practices; and (3) inform effective individualized interventions.

Finally, because poor dietary practices are linked to many chronic diseases and multiple factors influence adult dietary practices, identifying these barriers collectively at the individual level will ultimately determine if an increase in the number of barriers to healthy diet can predict increased risk for diet sensitive chronic diseases, therefore, inform personalized prevention interventions, particularly those aiming to prevent chronic diseases.

## Supplementary information

**Additional file 1:** Task instructions for expert reviewers. Letter to expert reviewers requesting their participation, describes the study background, frameworks and aim. The letter included a detail description and task instructions to expert panel.

**Additional file 2:** Task spreadsheet with list of identified items assigned to TDF domains. Excel spreadsheet containing the task performed by expert reviewers.

**Additional file 3:** Table 5 Presents 12 validated domains. All validated items selected from NHANES 2011–2012 database written in their complete form.

## Data Availability

The data and material supporting the conclusions of this article are included within the article and its additional files.

## Abbreviations

BCW: Behavior Change Wheel
COM-B: Capability, Opportunity, Motivation and Behavior
DHS: Dietary Health Status
NHANES: National Health and Nutrition Examination Surveys
TDF: Theoretical Domains Framework
