# Supplementary material for: Establishing content validity for a conceptualized instrument to measure barriers to eating a healthful diet in adults: a consensus approach
Source: BMC Health Serv Res. 2020 Jan 16;20:41. doi: 10.1186/s12913-020-4890-7 (PMC6966857; doi:10.1186/s12913-020-4890-7)
Supplement: Supplementary file 1 — Additional file 1: Task instructions for expert reviewers. Letter to expert reviewers requesting their participation, describes the study background, frameworks and aim. The letter included a detail description and task instructions to expert panel. [file 12913_2020_4890_MOESM1_ESM.docx]

Additional **file 1: Task instructions for expert reviewers**

**OVERVIEW**

Dear expert reviewers,

My name is Enia Zigbuo-Wenzler, and I am a Ph.D. candidate in the College of Nursing at the Medical University of South Carolina. My research interest lies in the areas of food security and dietary risks and their association with diet sensitive diseases. My dissertation study is aimed at developing a tool/scale that is capable of assessing multi-dimensions of a person’s dietary risk. The study was approved by the Medical University of South Carolina (MUSC) College of Nursing.

You are being asked to participate as part of a panel of reviewers because of your expertise in health and/or nutritional practice. I am seeking your feedback on selected variable items obtained from the National Health and Nutrition Examination Survey (NHANES) 2011-2012 datasets to establish independent review of the variables that have been preliminarily assigned to 13 of the 14 theoretical domains that comprise the Theoretical Domains Framework [(TDF), see Table 1 in Appendix **A**]. This expert review process is being conducted to establish content validity for variables to include as items on the tool/scale that will be developed. The capacity of a tool/scale to accurately identify individuals with multiple dietary risks may have implications for policy, education and practice.

**Background**

The dietary practices of individuals in the United States (U.S.) are influenced by their life situation as well as individual, socio-cultural, and other contextual factors (HHS, 2015 and Darmon & Drewnowski, 2008). The relationship among these factors could affect one’s health (Darmon & Drewnowski, 2008), as the link between diet and health is well established. In addition, diet is also a major modifiable risk factor of chronic diseases (Murray et al., 2013; Yoon et al. 2014). Based on my clinical experience as a Family Nurse Practitioner, my central hypothesis is that multiple factors collectively influence one’s diet and increase one’s risk for diet-sensitive chronic diseases. Those influencing factors need to be identified and addressed collectively. Currently, I am unaware of any multidimensional tool/scale to assess dietary risks. Therefore, *the aim of this study is to develop a multidimensional tool/scale by combining and analyzing the validity of select variables from the 2011-2012 NHANES datasets to determine dietary risk.* It is crucial that evaluations of variable items are based on a theoretical framework that covers a full range of current scientific explanations for human behavior. Therefore, the TDF in conjunction with the hub of the Behavior Change Wheel (BCW) framework, the COM-B model (see (Appendix) Figure 1) were used to guide the systematic identification of variable items from NHANES 2011-2012 datasets.

**Frameworks**

The TDF combines behavioral change theories into one framework that is used to identify barriers relevant to behavior change and to design practical interventions to address them (Michie et al., 2005; Cane et al., 2012). The TDF comprises 14 domains representing barriers, and each domain has a set of theoretical constructs for a total of 84 variables, the following 14 domains are derived from multiple psychological and organizational behavior change theories: knowledge; skills, social/professional role and identity; beliefs about capabilities; optimism; beliefs about consequences; reinforcement; intentions; goals; memory, attention and decision processes; environmental context and resources; social influences; and emotion and behavioral regulation (Cane et al., 2012). Researchers have used the TDF to develop theory-based questionnaires to identify and understand potential factors influencing human behavior and to guide the design of effective interventions to address them (Huijg et al., 2014; Taylor et al., 2013; Beenstock et al., 2012)

The framework was developed by Michie and colleagues over the last decade and was refined in 2012 by Cane and colleagues. The TDF is outlined in **Table 2** in the Appendix **A**.

The BCW was formed by combining 19 behavior change frameworks identified in a systematic literature review to provide a comprehensive, coherent, and universal approach to guide researchers/developers when designing behavior change interventions (Michie et al, 2011). The framework consists of three main layers; however, because the focus of the current study is to identify intrinsic and extrinsic factors that influence a person’s dietary choices, only the framework’s hub, the COM-B, was used. The hub “behavior system,” referred to as the COM-B system, is used to identify behaviors that need to be understood and modified. The COM-B system recognizes that behavior is part of an interacting system involving three components: capability, opportunity, and motivation (Michie et al, 2011). The COM-B proposes that for behavior change to occur, the person performing the behavior needs to 1) have the physical and psychological **capability** to perform the behavior, 2) have the social and physical **opportunity** to perform the behavior, and 3) be **motivated** to perform the behavior. Therefore, the COM-B is used in this study to identify variable items that measure the sources that reflect influences on behavior, which may need to be understood and modified.

As illustrated, the theoretical domains have been mapped to specific behavior change techniques (BCTs) that are the active components of interventions related to each domain (Debono et al., 2017). The COM-B system presents the three conditions that are required for behavior change to occur. Both frameworks are interconnected because each domain of the TDF relates to a COM-B component. Together, the framework provides a theory-based systematic approach for identifying and mapping variable items from NHANES to consider as items on the proposed instrument. The linkage between the TDF and COM-B is illustrated in (Appendix) Figure 2.

**INSTRUCTIONS TO EXPERT PANEL**

Your participation in this study is sincerely appreciated. If you decide to participate as a reviewer, please reply to me of your acceptance within one week via email. Once you have agreed to participate, you will need to return the completed review within three weeks via email to zigbuoaw@musc.edu. However, if this timeline does not work for you, please let me know as soon as possible so we can establish a reasonable completion date. Please contact me, Enia Zigbuo-Wenzler (zigbuoaw@musc.edu), if you have any questions.

**Task Overview:**

Please read through the instructions carefully prior to completing the task. Definitions of terms are presented in **Table 3** in Appendix A to assist you in completing the task. The task will be performed in an Excel spreadsheet, “Zigbuo-wenzler_expert-review_task.” The spreadsheet has 9 columns and 14 color coded rows. The last two columns provide the available response options. Please download the file onto your desktop, open it, and save as “Zigbuo-wenzler_expert-review_task_ plus your initials (e.g. Zigbuo-wenzler_expert-review_task_EZ).” The spreadsheet is large and to avoid zooming in and out, please follow the instructions to fit the columns of the spreadsheet exactly to your screen to reduce the width so you can see all the columns. However, you will need to scroll down to see the end. Your task is to provide your expert opinion whether a variable item belongs to the domain it is currently assigned; **“yes” indicates the item** belongs to assigned domain category; **“no” indicates the item** does not belong to assigned domain category. If you select “no”, please re-assign the variable item to another domain category.

The last two columns, **H** (Yes/No selection option) and **I** (Domain re-assignment) provide a drop-down list of the available response options.

**SCALE YOUR EXCEL SPREADSHEET TO FIT YOUR SCREEN INSTRUCTIONS**

• Select columns A to I from the top of the spreadsheet.

• Choose View, Zoom, then select Fit Selection

• Click OK

**How to determine if the variable item is in the accurate domain category:**

**1.** **Determine if the variable item is in the accurate domain**

Please review the descriptions provided in columns **B-D** (TDF domain description, TDF theoretical construct) and Study researchers adapted domain description, respectively)**, F** (NHANES variable description), and **G** (NHANES variable item question) to assist you to determine whether the variable item column **E** (NHANES variable item) was appropriately assigned to the accurate domain.

**2. Choose a response option**

Please decide based on your expert opinion and the descriptions provided in columns **B-D, F,** and **G** whether each variable item is a measure of the domain it is currently assigned. Select “yes”, the item belongs to the domain category or “no”, the item does not belong to the current domain using the dropdown menu response option in column **H**.

- Go to column **H** to select your response from a drop-down list of **Yes/No** option for each item.
- Click in the variable item cell in column **H,** a down arrow to the right of the cell will appear.
- Click on the down arrow, a list of **Yes/No** option will appear.
- Scroll to your response choice, the one that is highlighted. Click on it to select, it will appear in the cell.
- Once you are happy with your selection, please move to the next variable item.

**How to re-assign the variable item to another domain category:**

If you select “no” that the variable item does not belong to the domain it is currently assigned, please re-assign it to another domain category. You may re-assign a variable item only to one other domain category.

**1**. **Re-assigning a variable item another domain category**

Based on your expert opinion, please indicate another domain category for the variable item you rated “no” by selecting from the dropdown list of 14 domain options provided in column **I**.

**2. Choose a response option**

- Go to column **I** to select your response from a drop-down list of **14 domain** options for each item.
- Click in the variable item cell in column **I, and** a down arrow to the right of the cell will appear.
- Click on the down arrow, and a list of 14 domains will appear.
- Scroll to your response choice, the one that is highlighted. Click on it to select, and it will appear in the cell.
- Once you are satisfied with your selection, please move to the next variable item.

After receiving all completed tasks, the study team will review your responses and address any re-assignment of variable items to another domain. We may have a few follow-up questions and will contact you via email.

Thank you for participating on this expert panel review. Your expertise and time is appreciated through this critical process of my dissertation. My dissertation chair is Dr. Gayenell Magwood (magwoodg@musc.edu), and committee members are Drs. Martina Mueller and Angela Fraser.

Sincerely,

Enia Zigbuo-Wenzler, APRN, MSN, BC-FNP, MPH

PhD Candidate

Medical University of South Carolina/College of Nursing

Email: [zigbuoaw@musc.edu](mailto:zigbuoaw@musc.edu)

**REFERENCES**

1. Beenstock, J., Sniehotta, F. F., White, M., Bell, R., Milne, E. M., & Araujo-Soares, V. (2012). What helps and hinders midwives in engaging with pregnant women about stopping smoking? A cross-sectional survey of perceived implementation difficulties among midwives in the North East of England. Implementation Science, 7(1), 36. doi: 10.1186/1748-5908-7-36
2. Cane, J., O’Connor, D., & Michie, S. (2012). Validation of the theoretical domains framework for use in behaviour change and implementation research. Implementation Science, 7(1), 37. doi: 10.1186/1748-5908-7-37
3. Center for Health Statistics (NCHS), Division of Health and Nutrition Examination Surveys (DHNES)a. 2013-2014 National Health and Nutrition Examination Survey (NHANES). Retrieved from https://www.cdc.gov/nchs/nhanes/nhanes2013-2014/overview_h.htm.
4. Darmon, N., & Drewnowski, A. (2008). Does social class predict diet quality? The American Journal of Clinical Nutrition, 87(5), 1107-1117.
5. Debono, D., Taylor, N., Lipworth, W., Greenfield, D., Travaglia, J., Black, D., & Braithwaite, J. (2017). Applying the Theoretical Domains Framework to identify barriers and targeted interventions to enhance nurses’ use of electronic medication management systems in two Australian hospitals. Implementation Science, 12(1), 42. doi: 10.1186/s13012-017-0572-1
6. Huijg, J. M., Gebhardt, W. A., Dusseldorp, E., Verheijden, M. W., Zouwe, N. v. d., Middelkoop, B. J., & Crone, M. R. (2014). Measuring determinants of implementation behavior: psychometric properties of a questionnaire based on the theoretical domains framework. Implementation Science, 9(33), 1-15
7. Michie, S., van Stralen, M., & West, R. (2011). The Behaviour Change Wheel: a new method for characterising and designing behaviour change interventions. Implement Sci, 6. doi: 10.1186/1748-5908-6-42
8. Michie, S., Johnston, M., Abraham, C., Lawton, R., Parker, D., & Walker, A. (2005). Making psychological theory useful for implementing evidence based practice: a consensus approach. Qual Saf Health Care, 14. doi: 10.1136/qshc.2004.011155
9. Murray C.J.L., Abraham, J., Ali, M.K., Alvarado, M., Atkinson, C., Baddour, L.M.,…Lopez, A.D. (2013). "The state of US health, 1990-2010: Burden of diseases, injuries, and risk factors." Journal of the American Medical Association, 310, s178-s179
10. Shepherd R. (1999). Social determinants of food choice. Proceedings of the Nutrition Society, 58, 807-812
11. Taylor, N., Lawton, R., & Conner, M. (2013). Development and initial validation of the determinants of physical activity questionnaire. Int J Behav Nutr Phys Act, 10. doi: 10.1186/1479-5868-10-74
12. United States Department of Health and Human Services (HHS) and U.S. Department of Agriculture (USDA) (2015). Chapter 2, Shifts needed to align with healthy eating patterns. Current eating patterns in the United States. 2015 – 2020 Dietary guidelines for Americans. 8th Edition. Retrieved from https://health.gov/dietaryguidelines/2015/guidelines/chapter-2/current-eating-patterns-in-the-united-states/
13. Yoon, P.W., Bastian, B., Anderson, R.N., Collins, J. L., & Jaffe, H. W. (2014). Potentially preventable deaths from the five leading causes of death — united states, 2008–2010. Centers for disease control and prevention (CDC), Morbidity and Mortality Weekly Report (MMWR). 63(17), 369-374

**Appendix A**

**(Appendix) Table 1: Theoretical Domains Framework (TDF) 14 theoretical domains, domain description, and the 84 theoretical constructs**

| Theoretical domain | Cane et al. domain description | Theoretical construct | |
| --- | --- | --- | --- |
| 1. Knowledge | An awareness of the existence of something | | 1. Knowledge (including knowledge of condition /scientific rationale) 2. Procedural knowledge 3. Knowledge of task environment |
| 1. Skills | An ability or proficiency acquired through practice | | 1. Skills 2. Skills development 3. Competence 4. Ability 5. Interpersonal skills 6. Practice 7. Skill assessment |
| 1. Social/professional role and identity | A coherent set of behaviors and displayed personal qualities of an individual in a social or work setting | | 1. Professional identity 2. Professional role 3. Social identity 4. Identity 5. Professional boundaries 6. Professional confidence 7. Group identity 8. Leadership 9. Organizational commitment |
| 1. Beliefs about capabilities | Acceptance of the truth, reality, or validity about an ability, talent | | 1. Self-confidence 2. Perceived competence 3. Self-efficacy 4. Perceived behavioral control 5. Beliefs 6. Self-esteem 7. Empowerment 8. Professional confidence |
| 1. Optimism | The confidence that things will happen for the best | | 1. Optimism 2. Pessimism 3. Unrealistic optimism 4. Identity |
| 1. Beliefs about consequences | Acceptance of the truth, reality, or validity about outcomes of a behavior in a given situation | | 1. Beliefs 2. Outcome expectancies 3. Characteristics of outcome expectancies 4. Anticipated regret 5. Consequents |
| 1. Reinforcement | Increasing the probability of a response by arranging a dependent relationship, or contingency | | 1. Rewards (proximal/distal, valued/not valued, probable/improbable) 2. Incentives 3. Punishment 4. Consequents 5. Reinforcement 6. Contingencies 7. Sanctions |
| 1. Intentions | A conscious decision to perform a behavior or a resolve to act in a certain way | 1. Stability of intentions 2. Stages of change model 3. Trans theoretical model and stages of change | |
| 1. Goals | Mental representation of outcomes or end states | 1. Goals (distal/proximal) 2. Goal priority 3. Goal/target setting 4. Goals (autonomous/controlled) 5. Action planning 6. Implementation intention | |
| 1. Memory, attention and decision processes | The ability to retain information, focus selectively on aspects of the environment, and choose between two or more alternatives | 1. Memory 2. Attention 3. Attention control 4. Decision making 5. Cognitive overload/tiredness | |
| 1. Environmental context and resources | Any circumstance of a person’s situation or environment that discourages or encourages the development of skills and abilities, independence, social competence | 1. Environmental stressors 2. Resources/material resources 3. Organizational culture /climate 4. Salient events/critical incidents 5. Person x environment interaction 6. Barriers and facilitators | |
| 1. Social influences | Those interpersonal processes that can cause an individual to change their thoughts, feelings, or behaviors | 1. Social pressure 2. Social norms 3. Group conformity 4. Social comparisons 5. Group norms 6. Social support 7. Power 8. Intergroup conflict 9. Alienation 10. Group identity 11. Modelling | |
| 1. Emotion | A complex reaction pattern, involving experiential, behavioral, and physiological elements, by which the individual attempts to deal with a personally significant matter or event | 1. Fear 2. Anxiety 3. Affect 4. Stress 5. Depression 6. Positive/negative affect 7. Burn-out | |
| 1. Behavioral regulation | Anything aimed at managing or changing objectively observed or measured actions | 1. Self-monitoring 2. Breaking habit 3. Action planning | |


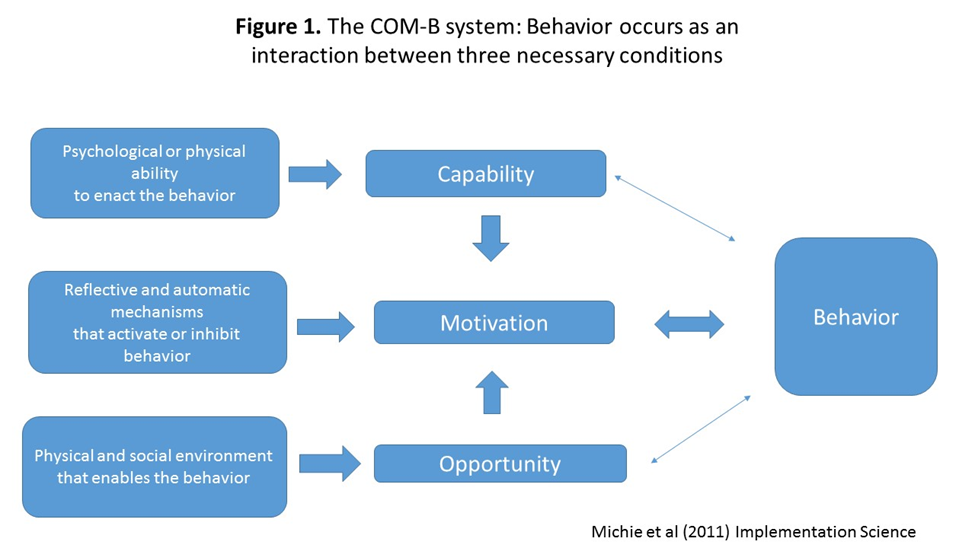


**(Appendix) Figure 1: The COM-B system: Behavior occurs as an interaction between three necessary conditions**


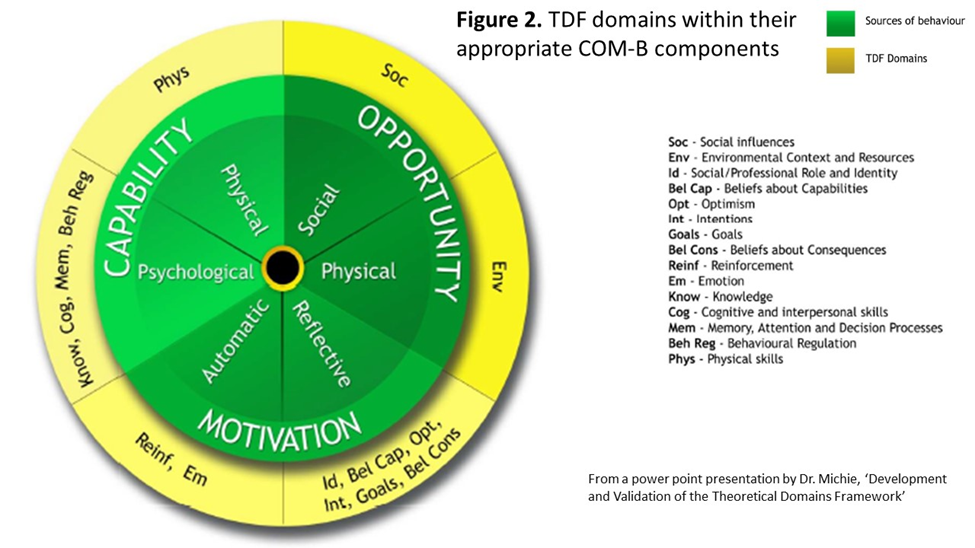


**(Appendix) Figure 2: TDF domains within their appropriate COM-B components**

**(Appendix) Table 2: 14 TDF domains, their TDF description and study researchers’ operational description**

| **TDF Theoretical Domain** | **TDF Domain Description** | **Study Researchers Operational Description** |
| --- | --- | --- |
| Knowledge | An awareness of the existence of something | An awareness of the dietary guidelines, their general health and health risks factors and the benefits of sports and recreational activities |
| Beliefs about capabilities | Acceptance of the truth, reality, or validity about an ability, talent, or facility that a person can put to constructive use | A person’s belief concerning their confidence, control, or performance concerning making appropriate dietary choices, staying healthy and engaging in sports and recreational activities |
| Beliefs about consequences | Acceptance of the truth, reality, or validity about outcomes of a behavior in a given situation | A person’s subjective rating of his/her general health, diet, and weight and his/her belief about the outcomes of making appropriate dietary choices, staying healthy and engaging in sports and recreational activities |
| Reinforcement | Increasing the probability of a response by arranging a dependent relationship, or contingency, between the response and a given stimulus | Internal or external responses to a person's behavior that affect the likelihood of making appropriate dietary choices, staying healthy and engaging in sports, fitness and recreational activities [Social Cognitive Theory (SCT)] |
| Memory, attention and decision processes | The ability to retain information, focus selectively on aspects of the environment, and choose between two or more alternatives | The ability to retain information concerning diet and health and to be able to focus on making appropriate dietary and health choices |
| Environmental context and resources | Any circumstance of a person’s situation or environment that discourages or encourages the development of skills and abilities, independence, social competence, and adaptive behavior | Any characteristics of the socio-political context, organization, and the person that discourages or encourages a person to make appropriate dietary choices, stay healthy and engage in sports and recreational activities |
| Social influences | Those interpersonal processes that can cause an individual to change their thoughts, feelings, or behaviors | A person’s association with people and situations in society that dictates the way he/she thinks about things that might affect his/her diet, health, and sports and recreational activity level |
| Behavioral regulation | Anything aimed at managing or changing objectively observed or measured actions | All the things a person does concerning their diet, health and sports and recreational activities |
| Optimism | The confidence that things will happen for the best, or that desired goals will be attained | A person’s confidence that things will happen for the best; never give up hope or look at the bright side of life |
| Emotion | A complex reaction pattern, involving experiential, behavioral, and physiological elements, by which the individual attempts to deal with a personally significant matter or event | A subjective psychophysiological experience that might affect a person’s likelihood of making appropriate dietary and health choices, and engaging in sports and recreational activities |
| Skills | An ability or proficiency acquired through practice | The competence or capacity that help a person routinely manage their diet and health in a productive manner; making appropriate dietary choices, staying healthy, and engaging in sports and recreational activities |
| Social/professional role and identity | A coherent set of behaviors and displayed personal qualities of an individual in a social or work setting | A coherent set of dietary and health promotion behaviors and displayed personal qualities of an individual in a social setting |
| Intentions | A conscious decision to perform a behavior or a resolve to act in a certain way | Readiness/commitment to make healthy dietary choices, stay healthy and engage in sports and recreational activities |
| Goals | Mental representation of outcomes or end states that an individual wants to achieve | An aim or an objective a person wants to achieve concerning their diet and health |

**(Appendix) Table 3: Definitions/terms that may assist you during task completion**

| Term | Definition |
| --- | --- |
| Theoretical Domains Framework (TDF) | A combination of behavioral change theories into one framework that may use to identify the sources relevant to behavior change. The TDF comprises of 14 domains and 84 constructs (Cane et al., 2012). Revision version 2012. |
| Theoretical domains (domain) | A group of related theoretical constructs (Michie et al., 2005 and Cane et al., 2012). |
| Theoretical construct (construct) | A concept specially devised to be part of a theory (Michie et al., 2005 and Cane et al., 2012). |
| TDF domain description. | Each domain as defined by the TDF researchers. |
| Authors’ domain description | Each domain as defined by this study’s authors based on the TDF researchers’ definition of theoretical domains and construct and as applicable to this study. |
| National Health and Nutrition Examination Survey (NHANES) variable item | Variable items obtained from the 2011-2012 NHANES datasets based on the TDF domain and constructs and the COM-B model. |
| NHANES variable item question | The exact questions used my NHANES’s interviewers for each variable item. |
