# Supplementary material for: Establishing content validity for a conceptualized instrument to measure barriers to eating a healthful diet in adults: a consensus approach
Source: BMC Health Serv Res. 2020 Jan 16;20:41. doi: 10.1186/s12913-020-4890-7 (PMC6966857; doi:10.1186/s12913-020-4890-7)
Supplement: Supplementary file 3 — Additional file 3: Table 5 Presents 12 validated domains. All validated items selected from NHANES 2011–2012 database written in their complete form. [file 12913_2020_4890_MOESM3_ESM.docx]

| **Final Items in their Domain** | | | |
| --- | --- | --- | --- |
| **TDF theoretical domain** | **TDF domain description** | **Study's researchers adapted domain description** | **NHANES item question** |
| **Knowledge** | An awareness of the existence of something | An awareness of the dietary guidelines, their general health and health risks factors and the benefits of sports and recreational activities | Next I’m going to ask a few questions about the nutritional guidelines recommended for Americans by the federal government. {Have you} heard of My Pyramid? |
|  |  |  | {Have you} heard of the Food Pyramid or the Food Guide Pyramid? |
| **Beliefs about capabilities** | Acceptance of the truth, reality, or validity about an ability, talent, or facility that a person can put to constructive use | A person’s belief concerning their confidence, control, or performance concerning making appropriate dietary choices, staying healthy and engaging in sports and recreational activities | How difficult have these problems made it for you to do your work, take care of things at home, or get along with people? |
|  |  |  | [Over the last 2 weeks, how often have you been bothered by the following problems:] feeling bad about yourself - or that you are a failure or have let yourself or your family down? |
| **Beliefs about consequences** | Acceptance of the truth, reality, or validity about outcomes of a behavior in a given situation | A person’s subjective rating of his/her general health, diet, and weight and his/her belief about the outcomes of making appropriate dietary choices, staying healthy and engaging in sports and recreational activities | {First} I have some general questions about {your} health. Would you say {your} health in general is excellent, very good, good, fair, or poor? |
|  |  |  | Next I have some questions about {your} eating habits. In general, how healthy is {your} overall diet; excellent, very good, good, fair, or poor? |
|  |  |  | {Do you} consider {your} self now to be; overweight, underweight or about the right weight? |
|  |  |  | Would {you} like to weigh more, less or same? |
|  |  |  | How much {would you} like to weigh? |
| **Reinforcement** | Increasing the probability of a response by arranging a dependent relationship, or contingency, between the response and a given stimulus | Internal or external responses to a person's behavior that affect the likelihood of making appropriate dietary choices, staying healthy and engaging in sports, fitness and recreational activities [Social Cognitive Theory (SCT)] | To lower {your} risk for certain diseases, during the past 12 months {have you} ever been told by a doctor or health professional to: control {your} weight or lose weight? |
|  |  |  | To: increase {your} physical activity or exercise? |
|  |  |  | To: reduce the amount of sodium or salt in {your} diet? |
|  |  |  | To: reduce the amount of fat or calories in {your} diet? |
|  |  |  | Because of {your} (high blood pressure/hypertension), {have you} ever been told to . . . take prescribed medicine? |
|  |  |  | Did a doctor or other health professional tell {you} to take {your} blood pressure at home? |
|  |  |  | [To lower (your) blood cholesterol, (have) (you) ever been told by a doctor or other health professional]... to take prescribed medicine? |
| **Memory, attention and decision processes** | The ability to retain information, focus selectively on aspects of the environment, and choose between two or more alternatives | The ability to retain information concerning diet and health and to be able to focus on making appropriate dietary and health choices | [Over the last 2 weeks, how often have you been bothered by the following problems: feeling tired or having little energy? |
|  |  |  | [Over the last 2 weeks, how often have you been bothered by the following problems:] trouble concentrating on things, such as reading the newspaper or watching TV? |
|  |  |  | {Are you} limited in any way because of difficulty remembering or because {you} experience{s} periods of confusion? |
| **Environmental context and resources** | Any circumstance of a person’s situation or environment that discourages or encourages the development of skills and abilities, independence, social competence, and adaptive behavior | Any characteristics of the socio-political context, organization, and the person that discourages or encourages a person to make appropriate dietary choices, stay healthy and engage in sports and recreational activities | What is the ratio of your family income to poverty? |
|  |  |  | For the last 12 months, what has been your household food security status? |
|  |  |  | In the last 12 months, did {you/you or any member of your household} receive SNAP or Food Stamp benefits? |
|  |  |  | In the last 12 months, did (you/you or any member of your household) ever get emergency food from a church, a food pantry, or a food bank, or eat in a soup kitchen? |
|  |  |  | In this part of the survey I will ask you questions about {your} work experience. Which of the following {were you} doing last week. |
|  |  |  | What is the highest grade or level of school {you have} completed or the highest degree {you have} received? |
|  |  |  | During the past 30 days, on the days that {you/SP} smoked, how many cigarettes did {you/s/he} smoke per day? |
|  |  |  | The next questions are about how much money {your family spends/you spend} on food. During the past 30 days, how much money {did your family/did you} spend at supermarkets or grocery stores? Please include purchases made with food stamps. |
|  |  |  | About how much money {did your family/did you} spend on food at these types of stores? (Please do not include any stores you have already told me about.) |
|  |  |  | During the past 30 days, how much money {did your family/did you} spend on eating out? Please include money spent in cafeterias at work or at school or on vending machines, for all family members. |
|  |  |  | During the past 30 days, how much money {did your family/did you} spend on food carried out or delivered? Please do not include money you have already told me about. |
| **Social influences** | Those interpersonal processes that can cause an individual to change their thoughts, feelings, or behaviors | A person’s association with people and situations in society that dictates the way he/she thinks about things that might affect his/her diet, health, and sports and recreational activity level | In what country {were you} born? Born in 50 US states or Washington, DC |
|  |  |  | Length of time the participant has been in the US. |
|  |  |  |  |
|  |  |  | Please look at the categories on this card. What race or races {do you} consider {yourself} to be? Please select one or more. |
|  |  |  | Gender of the participant |
|  |  |  | {Are you} now married, widowed, divorced, separated, never married or living with a partner? |
|  |  |  | {Are you} a citizen of the United States? |
|  |  |  | Age in years of the participant at the time of screening. Individuals 80 and over are top coded at 80 years of age. |
|  |  |  | Total number of people in the Family |
|  |  |  | What language(s) {do you} usually speak at home? THIS VARIABLE ONLY APPLY TO PARTICIPANTS SELF-IDENTIFIED AS "NON-HISPANIC WHITE", "NON-HISPANIC BLACK", OR "OTHER RACE - INCLUDING MULTI-RACIAL" (i.e., RIDRETH3=3, 4, OR 7). |
|  |  |  | What language(s) {do you} usually speak at home? THIS VARIABLE ONLY APPLY TO PARTICIPANTS SELF-IDENTIFIED AS "NON-HISPANIC WHITE", "NON-HISPANIC BLACK", OR "OTHER RACE - INCLUDING MULTI-RACIAL" (i.e., RIDRETH3=3, 4, OR 7). |
|  |  |  | What language(s) {do you} usually speak at home? THIS VARIABLE ONLY APPLY TO PARTICIPANTS SELF-IDENTIFIED AS "NON-HISPANIC WHITE", "NON-HISPANIC BLACK", OR "OTHER RACE - INCLUDING MULTI-RACIAL" (i.e., RIDRETH3=3, 4, OR 7). |
|  |  |  | Now I'm going to ask you about language use. What language(s) {do you} usually speak at home? {Do you} speak only Spanish, more Spanish than English, both equally, more English than Spanish, or only English? THIS VARIABLE ONLY APPLY TO PARTICIPANTS SELF-IDENTIFIED AS "MEXICAN AMERICAN" OR "OTHER HISPANIC" (i.e., RIDRETH3=1, OR 2). |
|  |  |  | {Do you} speak only (NON-ENGLISH LANGUAGE), more (NON-ENGLISH LANGUAGE) than English, both equally, more English than (NON-ENGLISH LANGUAGE), or only English? THIS VARIABLE ONLY APPLY TO PARTICIPANTS SELF-IDENTIFIED "NON-HISPANIC ASIAN" (i.e., RIDRETH3=6). |
| **Behavioral regulation** | Anything aimed at managing or changing objectively observed or measured actions | All the things a person does concerning their diet, health and sports and recreational activities | In a typical week, on how many days {do you} do vigorous-intensity sports, fitness or recreational activities? |
|  |  |  | {Do you} do any vigorous-intensity sports, fitness, or recreational activities that cause large increases in breathing or heart rate like running or basketball for at least 10 minutes continuously? |
|  |  |  | Do you} do any moderate-intensity sports, fitness, or recreational activities that cause a small increase in breathing or heart rate such as brisk walking, bicycling, swimming, or golf for at least 10 minutes continuously? |
|  |  |  | Are you currently on any kind of diet, either to lose weight or for some other health-related reason? (if Yes, which diet) |
|  |  |  | {Did you} take {your} blood pressure at home during the last 12 months? |
|  |  |  | How much time {do you} spend doing vigorous-intensity sports, fitness or recreational activities on a typical day? |
|  |  |  | In a typical week, on how many days {do you} do moderate-intensity sports, fitness or recreational activities? |
|  |  |  | How much time {do you} spend doing moderate-intensity sports, fitness or recreational activities on a typical day? |
|  |  |  | Are you currently on any kind of diet, either to lose weight or for some other health-related reason? What kind of diet are you on? Is it a weight loss or low calorie diet: low fat or cholesterol diet; low salt or sodium diet; sugar free or low sugar diet; low fiber diet; high fiber diet; diabetic diet; or another type of diet?) |
|  |  |  | Are you currently on any kind of diet, either to lose weight or for some other health-related reason? What kind of diet are you on? Is it a weight loss or low calorie diet: low fat or cholesterol diet; low salt or sodium diet; sugar free or low sugar diet; low fiber diet; high fiber diet; diabetic diet; or another type of diet?) |
|  |  |  | Are you currently on any kind of diet, either to lose weight or for some other health-related reason? What kind of diet are you on? Is it a weight loss or low calorie diet: low fat or cholesterol diet; low salt or sodium diet; sugar free or low sugar diet; low fiber diet; high fiber diet; diabetic diet; or another type of diet?) |
|  |  |  | Are you currently on any kind of diet, either to lose weight or for some other health-related reason? What kind of diet are you on? Is it a weight loss or low calorie diet: low fat or cholesterol diet; low salt or sodium diet; sugar free or low sugar diet; low fiber diet; high fiber diet; diabetic diet; or another type of diet?) |
|  |  |  | Are you currently on any kind of diet, either to lose weight or for some other health-related reason? What kind of diet are you on? Is it a weight loss or low calorie diet: low fat or cholesterol diet; low salt or sodium diet; sugar free or low sugar diet; low fiber diet; high fiber diet; diabetic diet; or another type of diet?) |
|  |  |  | Are you currently on any kind of diet, either to lose weight or for some other health-related reason? What kind of diet are you on? Is it a weight loss or low calorie diet: low fat or cholesterol diet; low salt or sodium diet; sugar free or low sugar diet; low fiber diet; high fiber diet; diabetic diet; or another type of diet?) |
|  |  |  | Are you currently on any kind of diet, either to lose weight or for some other health-related reason? What kind of diet are you on? Is it a weight loss or low calorie diet: low fat or cholesterol diet; low salt or sodium diet; sugar free or low sugar diet; low fiber diet; high fiber diet; diabetic diet; or another type of diet?) |
|  |  |  | Are you currently on any kind of diet, either to lose weight or for some other health-related reason? What kind of diet are you on? Is it a weight loss or low calorie diet: low fat or cholesterol diet; low salt or sodium diet; sugar free or low sugar diet; low fiber diet; high fiber diet; diabetic diet; or another type of diet?) |
|  |  |  | Are you currently on any kind of diet, either to lose weight or for some other health-related reason? What kind of diet are you on? Is it a weight loss or low calorie diet: low fat or cholesterol diet; low salt or sodium diet; sugar free or low sugar diet; low fiber diet; high fiber diet; diabetic diet; or another type of diet?) |
|  |  |  | Are you currently on any kind of diet, either to lose weight or for some other health-related reason? What kind of diet are you on? Is it a weight loss or low calorie diet: low fat or cholesterol diet; low salt or sodium diet; sugar free or low sugar diet; low fiber diet; high fiber diet; diabetic diet; or another type of diet?) |
|  |  |  | Are you currently on any kind of diet, either to lose weight or for some other health-related reason? What kind of diet are you on? Is it a weight loss or low calorie diet: low fat or cholesterol diet; low salt or sodium diet; sugar free or low sugar diet; low fiber diet; high fiber diet; diabetic diet; or another type of diet?) |
|  |  |  | During the past 7 days, how many meals {did you} get that were prepared away from home in places such as restaurants, fast food places, food stands, grocery stores, or from vending machines? |
|  |  |  | How many of those meals {did you/} get from a fast-food or pizza place? |
|  |  |  | Some grocery stores sell “ready to eat” foods such as salads, soups, chicken, sandwiches and cooked vegetables in their salad bars and deli counters. During the past 30 days, how often did {you} eat “ready to eat” foods from the grocery store? Please do not include sliced meat or cheese you buy for sandwiches and frozen or canned foods. |
|  |  |  | During the past 30 days, how often did {you} eat frozen meals or frozen pizzas? |
|  |  |  |  |
|  |  |  | To lower {your} risk for certain diseases, {are you} now doing any of the following: controlling {your} weight or losing weight? |
|  |  |  | To lower {your} risk for certain diseases, {are you} now doing any of the following: increasing {your} physical activity or exercise? |
|  |  |  | To lower {your} risk for certain diseases, {are you} now doing any of the following: reducing the amount of sodium or salt in {your} diet? |
|  |  |  | To lower {your} risk for certain diseases, {are you} now doing any of the following: reducing the amount of fat or calories in {your} diet? |
|  |  |  | {Have you} looked up the My Pyramid plan for a {man/woman/person} {your} age on the internet? |
|  |  |  | {Have you} tried to follow the {My Pyramid Plan/Pyramid plan} recommended for {you}? |
|  |  |  | During the past 12 months, {have you} tried to lose weight? Tried to lose weight in past year. |
|  |  |  | How did {you} try to lose weight? Ate less to lose weight. |
|  |  |  | How did {you/SP} try to lose weight? Switched to foods with lower calories. |
|  |  |  | How did {you/SP} try to lose weight? Ate less fat to lose weight. |
|  |  |  | How did {you/SP} try to lose weight? Exercised to lose weight. |
|  |  |  | How did {you/SP} try to lose weight? Skipped meals. |
|  |  |  | How did {you/SP} try to lose weight? Ate diet foods or products. |
|  |  |  | How did {you/SP} try to lose weight? Used a liquid diet formula. |
|  |  |  | How did {you/SP} try to lose weight? Joined a weight loss program. |
|  |  |  | How did {you/SP} try to lose weight? Took prescription diet pills. |
|  |  |  | How did {you/SP} try to lose weight? Took non-RX suppl. to lose weight. |
|  |  |  | How did {you/SP} try to lose weight? Took laxatives or vomited. |
|  |  |  | How did {you/SP} try to lose weight? Drank a lot of water. |
|  |  |  | How did {you/SP} try to lose weight? Followed a special diet. |
|  |  |  | How did {you/SP} try to lose weight? Ate fewer carbohydrates. |
|  |  |  | How did {you/SP} try to lose weight? Started to smoke or began to smoke again. |
|  |  |  | How did {you/SP} try to lose weight? Ate more fruits, vegetables, salads. |
|  |  |  | How did {you/SP} try to lose weight? Changed eating habits. |
|  |  |  | How did {you/SP} try to lose weight? Ate less sugar, candy, sweets. |
|  |  |  | How did {you/SP} try to lose weight? Ate less junk food or fast food. |
|  |  |  | How did {you/SP} try to lose weight? Other. |
|  |  |  | In the past 12 months, {did you} seek help from a personal trainer, dietitian, nutritionist, doctor or other health professional to lose weight? |
|  |  |  | Was that a personal trainer? |
|  |  |  | Was that a dietitian? |
|  |  |  | Was that a nutritionist? |
|  |  |  | Was that a doctor? |
|  |  |  | Was that other health professional? |
|  |  |  | Was the change between {your} current weight and {your} weight a year ago intentional? |
|  |  |  | During the past 12 months, {have you} done anything to keep from gaining weight? |
|  |  |  | What did {you} do to keep from gaining weight? Ate less food |
|  |  |  | What did {you} do to keep from gaining weight? Switched to foods with lower calories. |
|  |  |  | What did {you} do to keep from gaining weight? Ate less fat. |
|  |  |  | What did {you} do to keep from gaining weight? Exercised. |
|  |  |  | What did {you} do to keep from gaining weight? Skipped meals. |
|  |  |  | What did {you} do to keep from gaining weight? Ate diet foods or products. |
|  |  |  | What did {you} do to keep from gaining weight? Used liquid diet formula. |
|  |  |  | What did {you} do to keep from gaining weight? Joined program to not gain weight. |
|  |  |  | What did {you} do to keep from gaining weight? Took prescription diet pills. |
|  |  |  | What did {you} do to keep from gaining weight? Took non-prescription diet pills. |
|  |  |  | What did {you} do to keep from gaining weight? Took laxatives or vomited. |
|  |  |  | What did {you} do to keep from gaining weight? Drank a lot of water. |
|  |  |  | What did {you} do to keep from gaining weight? Followed a special diet. |
|  |  |  | What did {you} do to keep from gaining weight? Ate fewer carbohydrates. |
|  |  |  | What did {you} do to keep from gaining weight? Started to smoke or began to smoke again. |
|  |  |  | What did {you} do to keep from gaining weight? Ate more fruits, vegetables, salads. |
|  |  |  | What did {you} do to keep from gaining weight? Changed eating habits. |
|  |  |  | What did {you} do to keep from gaining weight? Ate less sugar, candy, sweets. |
|  |  |  | What did {you} do to keep from gaining weight? Ate less junk food or fast food. |
|  |  |  | What did {you} do to keep from gaining weight? Other. |
|  |  |  | {Are you} now taking a prescribed medicine? |
|  |  |  | How often {did you check your} blood pressure at home during the last 12 months? |
|  |  |  | {Are you} now taking a prescribed medicine? |
|  |  |  | {Are you} now taking insulin |
|  |  |  | {Are you} now taking diabetic pills to lower {your} blood sugar? These are sometimes called oral agents or oral hypoglycemic agents. |
|  |  |  | Two 24-hour dietary recall interviews to estimate the total intake of food energy (calories), nutrients, and non-nutrient food components from foods and beverages; consumed during the 24-hour period prior to the interview. First dietary recall, in-person in the Mobile Examination Center (MEC)  Second interview, by telephone 3 to 10 days later |
|  |  |  | How long has it been since you last smoked marijuana or hashish at least once a month for one year? INSTRUCTIONS TO SP: Please enter the number of days, weeks, months, or years, then select the unit of time. Have you ever, even once, used marijuana or hashish? |
|  |  |  | How long has it been since you last smoked marijuana or hashish at least once a month for one year? INSTRUCTIONS TO SP: Please enter the number of days, weeks, months, or years, then select the unit of time. Have you ever, even once, used marijuana or hashish? |
|  |  |  | How long has it been since you last used cocaine, in any form? INSTRUCTIONS TO SP: Please enter the number of days, weeks, months, or years, then select unit of time. Have you ever, even once, used cocaine, in any form? |
|  |  |  | How long has it been since you last used cocaine, in any form? INSTRUCTIONS TO SP: Please enter the number of days, weeks, months, or years, then select unit of time. Have you ever, even once, used cocaine, in any form? |
|  |  |  | How long has it been since you last used heroin? INSTRUCTIONS TO SP: Please enter the number of days, weeks, months, or years, then select the unit of time. |
|  |  |  | How long has it been since you last used heroin? INSTRUCTIONS TO SP: Please enter the number of days, weeks, months, or years, then select the unit of time. |
|  |  |  | How long has it been since you last used methamphetamine? INSTRUCTIONS TO SP: Please enter the number of days, weeks, months, or years, then select the unit of time. |
|  |  |  | How long has it been since you last used methamphetamine? INSTRUCTIONS TO SP: Please enter the number of days, weeks, months, or years, then select the unit of time |
|  |  |  | In {your} entire life, {have you} had at least 12 drinks of any type of alcoholic beverage? |
|  |  |  | In any one year, {have you} had at least 12 drinks of any type of alcoholic beverage? By a drink, I mean a 12 oz. beer, a 5 oz. glass of wine, or one and a half ounces of liquor. |
|  |  |  | In the past 12 months, on those days that {you} drank alcoholic beverages, on the average, how many drinks did {you} have? (By a drink, I mean a 12 oz. beer, a 5 oz. glass of wine, or one and a half ounces of liquor.) |
| **Optimism** | The confidence that things will happen for the best, or that desired goals will be attained | A person’s confidence that things will happen for the best; never give up hope or look at the bright side of life | Over the last 2 weeks, how often have you been bothered by the following problems: little interest or pleasure in doing things? |
|  |  |  | Over the last 2 weeks, how often have you been bothered by the following problems: feeling down, depressed, or hopeless? |
|  |  |  | Over the last 2 weeks, how often have you been bothered by the following problems: Thoughts that you would be better off dead or of hurting yourself in some way? |
| **Emotion** | A complex reaction pattern, involving experiential, behavioral, and physiological elements, by which the individual attempts to deal with a personally significant matter or event | A subjective psychophysiological experience that might affect a person’s likelihood of making appropriate dietary and health choices, and engaging in sports and recreational activities | During the past 30 days, for about how many days {have you} felt worried, tense, or anxious? |
|  |  |  |  |
| **New Domains created** | | | |
| **TDF theoretical domain** | **TDF domain description** | **Study's researchers adapted domain description** | **NHANES variable item question** |
| **Health Identity** | N/A | A person sense of self/identity in view of a health characteristic that he/she may have to identify with or has identified with | {Have you} ever been told by a doctor or other health professional that {you} had hypertension, also called high blood pressure? |
|  |  |  | {Were you} told on 2 or more different visits that {you} had hypertension, also called high blood pressure? |
|  |  |  | {Have you} ever been told by a doctor or other health professional that {you have} high normal blood pressure, prehypertension or borderline hypertension? |
|  |  |  | {Have you} ever been told by a doctor or other health professional that {your} blood cholesterol level was high? |
|  |  |  | The next questions are about specific medical conditions {Have you} ever been told by a doctor or health professional that {you have} diabetes or sugar diabetes? |
|  |  |  | {Have you} ever been told by a doctor or other health professional that {you have} any of the following: prediabetes, impaired fasting glucose, impaired glucose tolerance, borderline diabetes or that {your} blood sugar is higher than normal but not high enough to be called diabetes or sugar diabetes? |
|  |  |  | Now thinking about {your} mental health, which includes stress, depression, and problems with emotions, for how many days during the past 30 days was {your} mental health not good? |
|  |  |  | {Are you} limited in any way in any activity because of a physical, mental or emotional problem? |
|  |  |  | Has a doctor or other health professional ever told {you} that {you were} overweight? |
|  |  |  | Has a doctor or other health professional ever told {you} that {you} . . .had congestive heart failure? |
|  |  |  | Has a doctor or other health professional ever told {you} that {you} . . .had coronary (kor-o-nare-ee) heart disease? |
|  |  |  | Has a doctor or other health professional ever told {you} that {you} . . .had angina (an-gi-na), also called angina pectoris? |
|  |  |  | Has a doctor or other health professional ever told {you} that {you} . . .had a heart attack (also called myocardial infarction (my-o-car-dee-al in-fark-shun))? |
|  |  |  | Has a doctor or other health professional ever told {you} that {you} . . .had a stroke? |
| **Functional Status** | N/A | Any functional limitations caused by long-term physical, mental, and emotional problems or illness that impact a person ability to make appropriate life choices and to engage in activities that promote a healthy lifestyle | The next set of questions is about limitations caused by any long-term physical, mental or emotional problem or illness. Because of a health problem, {do you} have difficulty walking without using any special equipment? |
|  |  |  | The next questions ask about difficulties {you/SP} may have doing certain activities because of a health problem. By "health problem" we mean any long-term physical, mental or emotional problem or illness {not including pregnancy}. By {yourself} and without using any special equipment, how much difficulty {do you} have . . .walking for a quarter of a mile [that is about 2 or 3 blocks]? |
|  |  |  | The next questions are about {your} recent health during the 30 days outlined on the calendar. Thinking about {your} physical health, which includes physical illness and injury, for how many days during the past 30 days was {your} physical health not good? |
|  |  |  | During the past 30 days, for about how many days did pain make it hard for {you} to do {your} usual activities, such as self-care, work, or recreation? |
|  |  |  | Over the last 2 weeks, how often have you been bothered by the following problems:] trouble falling or staying asleep, or sleeping too much? |
|  |  |  | [Over the last 2 weeks, how often have you been bothered by the following problems:] poor appetite or overeating? |
|  |  |  | Over the last 2 weeks, how often have you been bothered by the following problems:] moving or speaking so slowly that other people could have noticed? Or the opposite - being so fidgety or restless that you have been moving around a lot more than usual? |
|  |  |  | The next set of questions is about limitations caused by any long-term physical, mental or emotional problem or illness. Does a physical, mental or emotional problem now keep {you} from working at a job or business? |
|  |  |  | {Are you} limited in the kind or amount of work {you} can do because of a physical, mental or emotional problem? |
|  |  |  | Ask about difficulties {you} may have doing certain activities because of a health problem. any long-term physical, mental or emotional problem or illness. By {yourself} and without using any special equipment, how much difficulty {do you} have . . .managing {your} money [such as keeping track of {your} expenses or paying bills]? |
|  |  |  | By {yourself} and without using any special equipment, how much difficulty {do you/does SP} have . . . participating in social activities [visiting friends, attending clubs or meetings or going to parties]? |
|  |  |  | The next questions ask about difficulties {you/SP} may have doing certain activities because of a health problem. By "health problem" we mean any long-term physical, mental or emotional problem or illness {not including pregnancy}.By {yourself} and without using any special equipment, how much difficulty {do you/does SP} have . . .preparing {your} own meals? |
